# The Self-Fulfilling Process of Clinical Race Correction: The Case of Eighth Joint National Committee Recommendations

**DOI:** 10.1089/heq.2023.0064

**Published:** 2023-11-30

**Authors:** Leah C. Savage, Aaron Panofsky

**Affiliations:** Institute for Society and Genetics, University of California, Los Angeles, Los Angeles, California, USA.

**Keywords:** hypertension, race adjustment, JNC 8 clinical guideline, slavery hypertension hypothesis, ACE inhibitor, ALLHAT

## Abstract

There is growing attention to how unfounded beliefs about biological differences between racial groups affect biomedical research and health care, in part, through race adjustment in clinical tools. We develop a case study of the Eighth Joint National Committee (JNC 8)'s 2014 Evidence-Based Guideline for the Management of High Blood Pressure in Adults, which recommends a distinct initial hypertension treatment for Black versus nonblack patients. We analyze the historical context, study design, and racialized findings of the Antihypertensive and Lipid-Lowering Treatment to Prevent Heart Attack Trial (ALLHAT) that informed development of the guideline. We argue that ALLHAT's racialized outcomes emanated from a poor and artificial study design and analysis weakened by implicit assumptions about race as biological. We show that the acceptance and utilization of ALLHAT for race correction arises from its historical context within the “inclusion-and-difference paradigm” and its indication of the inefficacy of angiotensin-converting-enzyme inhibitors for Black patients, which follows from the enduring, yet, refuted slavery hypertension hypothesis. We demonstrate that the JNC 8 guideline displays the self-fulfilling process of racial reasoning: presuppositions about racial differences inform the design and interpretation of research, which then conceptually reinforce ideas about racial differences leading to differential medical treatment. We advocate for the abolition of race adjustment and the integration of structural competency, biocritical inquiry, and race-conscious medicine into biomedical research and clinical medicine to disrupt the use of race as a proxy for ancestry, environment, and social treatment and to address the genuine determinants of racialized disparities in hypertension.

## Introduction

Racial disparities in cardiovascular disease (CVD) and guidelines recommending distinct hypertension treatment by race are often cited to claim that racial groups are biologically real entities, that race should be taken as primary health information, and that the current push to acknowledge race as a social construction is “political correctness” run amok in medicine.^1^ At the same time, more and more physicians interested in medicine as a vehicle for social change and social equity and sympathetic to the idea and evidence that race is socially constructed are grappling with racialized clinical tools and guidelines. But aimed at providing patient-centered care, they worry that they may harm patients by ignoring race or disregarding race-adjusted clinical tools.

This paper focuses on one such example of race adjustment, hypertension management guidelines, which commonly assert that angiotensin-converting enzyme (ACE) inhibitors are not as efficacious and should not be prescribed as initial antihypertensive therapy for Black patients.^2,3^ The urgency around this dilemma has intensified as emerging data demonstrates that even when racialized hypertension treatment guidelines are widely used, they do not appear to reduce racial disparities in hypertension control.^4^

Furthermore, drug treatment outcomes seem to vary more within than between racial groups, demonstrated by a study comparing the blood pressure (BP) outcomes of Black and white hypertensives on ACE inhibitor monotherapy.^5^ The investigators argue, “Race differences in group blood pressure responses to monotherapy with ACE inhibitors are an inaccurate means of predicting the magnitude of BP-lowering with an ACE inhibitor in individual patients of either race because of the near-complete overlap of the BP response distributions of Blacks and whites, and because a significant portion of the race difference in BP response is attributable to factors that vary at the level of the individual.”^5^ Indeed, physicians cognizant of and focused on reducing demonstrated racial inequities in hypertension^6^ are correct to be skeptical of race-based medical recommendations as a mechanism for their mitigation.

This article is a theoretical analysis and conceptual critique of the Eighth Joint National Committee (JNC 8) Evidence-Based Guideline for the Management of High Blood Pressure in Adults. We examine the origins of distinct treatment guidelines that so many lean on as a paradigmatic case. In this case, we assert that the race-specific components of the JNC 8 guideline and their primary evidence basis, the Antihypertensive and Lipid-Lowering Treatment to Prevent Heart Attack Trial (ALLHAT), demonstrate racialized thinking that is cognitively and institutionally self-affirming rather than based in good evidence. We analyze five interlocking issues in ALLHAT's design and analysis and show that ALLHAT's racialized outcomes, as well as their uptake by JNC 8, were a product of the racial worldview (RWV).

We offer a historical analysis of the conception of a Black patient-specific physiology, which is rooted in the debunked but perpetuated slavery hypertension hypothesis and part and parcel of the inclusion and difference paradigm. Furthermore, we illustrate that this case study exemplifies a self-fulfilling prophecy of racial reasoning, in which assumptions about racial difference inform the design and interpretation of research which then serve to reinforce ideas about racial differences leading to differential medical treatment. Finally, we call for a rejection of racial essentialism in biomedical research and clinical treatment and an embrace of biocritical inquiry, structural competency, and race-conscious medicine, which aim to parse out and address the specific social and structural determinants of racial inequities in hypertension.

## Hypertension, Chronic Kidney Disease, and Treatment Drugs

Hypertension is defined as of 2017 by the American College of Cardiology and the American Heart Association as having systolic pressure greater than or equal to 130 mmHg and/or diastolic pressure greater than or equal to 80 mmHg.^3^ There are several commonly prescribed drugs to treat hypertension; this article will focus on the medications tested in ALLHAT: thiazide-type diuretics, calcium channel blockers (CCBs), and ACE inhibitors,^7^ each of which have different biological mechanisms.

Thiazide-type diuretics inhibit sodium and water reabsorption in the kidney, decreasing fluid volume to diminish BP.^8^ CCBs prevent the entrance of calcium into the cells of the heart and arteries, decreasing the strength of heart contractions and relaxing blood vessels to lower BP.^9^ ACE inhibitors work in the renin-angiotensin-aldosterone system (RAAS).^10^ Normally, after renin converts angiotensin to angiotensin I, the ACE converts angiotensin I to the vessel-narrowing angiotensin II (its active form). ACE inhibitors inhibit this ACE enzyme.^10^ Without ACE inhibition, angiotensin II increases BP by constricting the blood vessels, and impacts greater levels of aldosterone, causing more water and sodium reabsorption and increasing blood volume as well as pressure.^10^

Hypertension is seen in most patients with chronic kidney disease (CKD) and can both lead to and result from CKD, which is defined by decreased kidney function, measured through estimated glomerular filtration rate (eGFR), or kidney damage, often indicated by the presence of protein in the urine.^11^ As kidney function worsens, the RAAS is upregulated with increases in the release of renin and downstream products, which can lead to or worsen hypertension.^11,12^ Thus, ACE inhibitors and angiotensin receptor blockers (ARB), both of which work within the RAAS, or a combination of these drugs and CCBs and diuretics are commonly recommended as initial treatment for patients with hypertension who suffer from CKD.^11^

## Racialized Recommendations by JNC 8

The JNC 8 Evidence-Based Guideline for the Management of High Blood Pressure in Adults, published in 2014, and a major focus of this article, was aimed at offering evidence-based recommendations to clinicians for managing hypertension. The guideline follows the logic of other racialized clinical decision-making tools in distinguishing drug treatment recommendations for the “general nonblack” versus the “general Black population.”^2^

It specifies that while nonblack patients should be treated initially with a “thiazide-type diuretic, calcium channel blocker, angiotensin-converting enzyme inhibitor, or angiotensin receptor blocker (ARB)” according to recommendation six, Black patients should be treated initially only with a thiazide-type diuretic or CCB, based on recommendation seven.^2^ Through recommendation eight, which cites the African American Study of Kidney Disease and Hypertension Study,^13^ the guideline recommends that patients with CKD and hypertension, “regardless of race or diabetes status,” be treated with “an angiotensin-converting enzyme inhibitor (ACEI) or ARB to improve kidney outcomes.”^2^

Significantly, as previously described, ACE inhibitors operate in the RAAS. There is a common idea in biomedical research that Black folks are predisposed to low concentrations of renin, the enzyme that converts angiotensin to angiotensin I in the RAAS or RAS, and therefore decreased plasma renin activity (PRA).^14^ This concept of a Black patient-specific physiology, defined by high salt sensitivity and low plasma renin, is commonly cited as an explanation for why ACE inhibitors are not as effective for Black patients, since ACE inhibitors lower BP by inhibiting the enzyme that acts on angiotensin I, the product of renin.^3,15–17^ The historical underpinnings of this supposition will be delineated later. Although the JNC 8 guideline does not explicitly call upon this supposed racialized physiology, its recommendations, which assert ACE inhibitors are not an appropriate first-line therapy for Black patients, follow this same logic.

In terms of CKD, the upregulation of the RAAS (especially intrarenally) commonly seen with this disease may lead to the conclusion that Black individuals with CKD, whose renin levels are bolstered, become good candidates for drugs that inhibit the RAAS, including ACE inhibitors.^12,^^*^ Indeed, JNC 8 recommends ACE inhibitors for all patients with CKD, regardless of race. These conclusions are essential and will be expanded on later in the context of the guideline and study on which this article focuses.

## How ALLHAT Underpins the JNC 8 Guideline

The evidentiary basis for recommendation seven—which explicitly recommends a differential initial hypertension treatment based on race—comes from the outcomes of the ALLHAT.^2^ This article focuses on this trial, a National Institutes of Health (NIH) National Heart, Lung, and Blood Institute funded study which began in August of 1993 and was completed in March 2002. ALLHAT was groundbreaking at the time of its design in its comparison of classically used diuretics with the more novel CCB and ACE inhibitor drugs in terms of “fatal coronary heart disease (CHD) incidence” as well as other CVD events.^7^

ALLHAT initially defined its primary outcomes as “combined fatal CHD or nonfatal myocardial infarction” and its secondary outcomes as “all-cause mortality, fatal and nonfatal stroke, combined CHD (the primary outcome, coronary revascularization, or angina with hospitalization), and combined CVD (combined CHD, stroke, other treated angina, heart failure (HF) [fatal, hospitalized, or treated nonhospitalized], and peripheral arterial disease).”^18^ Ultimately, ALLHAT reported several significant race-specific results based on secondary outcomes pointing to the inefficacy of lisinopril, the ACE inhibitor, for Black patients. These results, including a decreased reduction of systolic blood pressure (SBP) and increased relative risk (RR) of stroke and combined CVD for Black patients using lisinopril, the ACE inhibitor, as compared to chlorthalidone, the diuretic, informed the JNC 8 recommendations.^17^

ALLHAT's influence based on a racialized subgroup analysis is unsurprising as its intentions were specifically racialized from its inception. In ALLHAT's Protocol, the third listed rationale for the ALLHAT study is the “Importance of Comparing Antihypertensive Agents in African-Americans.”^7^ Indeed, the Protocol articulates the disproportionate burden of hypertension for Black Americans and thus the ramifications of the study for Black folks. Focused on testing the more expensive CCBs and ACEIs against cheaper diuretics partially due to a dearth of comparative studies attentive to hypertension and CHD reduction, the Protocol also explicitly states that the cost consideration is important for Black patients.^7^

ALLHAT asserts that it is essential for Black patients, whom they posit as more often low-income with decreased access to medical care, to know whether cheaper drugs such as diuretics are as efficacious as CCBs and ACEIs.^7^ The end of the protocol states, “… given the relative lack of clinical trials information in African-Americans, they should be heavily represented in the current trial so that the results will be directly applicable to them. For these reasons, the population for this trial will be at least 55% African American,” demonstrating the principal investigators' hypothesis of the existence of racialized distinctions in drug efficacy and aim to include an ample number of Black participants to ensure that any such differences are produced.^7^

## Racialized Flaws in ALLHAT and JNC 8

We argue that the differential racial recommendations crafted by JNC 8 and informed by ALLHAT are shaped and discredited by five interlocking issues in ALLHAT's design and analysis and the incorporation of outcomes into JNC 8; namely (1) poorly defined categories/measures; (2) *post hoc* analysis and reformulation of the study question; (3) artificial study design; (4) overstatement of drug differences; and (5) selective uptake of evidence. Here, we are artificially simplifying and separating the different components for the purpose of accessibility.

First, ALLHAT used poorly defined categories and metrics. Although ALLHAT's outcomes were used to create racialized clinical recommendations, ALLHAT never defined its racial categories or what race was meant to encapsulate (in terms of genetics, environmental exposure, lifestyle, etc.). This point will be expanded on later. Furthermore, HF, one of the components of combined CVD, was likely variable in its diagnosis.^19–21^ It was “not a hard end-point” and there were “no standard, preset criteria for the diagnosis” or follow-up for HF as compared to other outcomes, including stroke and myocardial infarction; rather, “it was determined by the investigator with only a tiny minority of events undergoing independent adjudication.”^19–21^

Moreover, 90.2% of participants were undergoing treatment for hypertension before the study, and many were likely taking commonly prescribed diuretics. For participants who switched from diuretic therapy to a nondiuretic therapy such as ACE inhibition, there may have been some effects of withdrawal, including HF.^19^

Second, ALLHAT's ultimate analysis diverged sharply from its initial study question and used secondary outcomes to make its conclusions. ALLHAT's first goal was comparing amlodipine, a CCB, and lisinopril, an ACE inhibitor, with chlorthalidone, a diuretic (the standard at the time).^20^ In terms of primary outcomes and “all other hard mortality end points,” there were no statistical differences found; demonstrating that “the newer drugs were at least as effective in the all-important category of saving lives and saving heart attacks, as were diuretics” for all patients.^20^

Instead of publishing this conclusion, however, ALLHAT pivoted to formulate a new question regarding the optimal first-step therapy for hypertension.^20^ But ALLHAT was not designed to be a first-step study, because as mentioned before, 90.2% of all participants were already taking hypertension medication, with no “washout” period before starting the study drug.^20^ For these patients, there was also no record of the length of consumption of the previous drug or consideration of BPs before the start of any medication.^20^ One critique went so far as to argue that this design of the study “precludes the possibility of answering the newly formulated research question.”^21^

Furthermore, ALLHAT applied its secondary end-points, especially RRs of stroke and combined CVD (which included HF and stroke outcomes), to produce the racialized conclusions used by JNC 8, although secondary end-points are normally “collected mainly for hypothesis generation and the elucidation of possibilities for further studies.”^20^ Indeed, there were no significant racialized differences found for the primary outcomes of combined fatal CHD or nonfatal myocardial infarction for any drugs.

Third, ALLHAT's study design was artificial and not aligned with clinical applications of the study's drugs.^14^ Diuretic usage in the lisinopril group was prohibited if BP goals were not met. Instead, ALLHAT enforced usage of the beta-blocker atenolol or clonidine or reserpine in combination with the ACE inhibitor lisinopril.^20^ The allowance of only these add-ons, which also suppress renin, compared to CCBs or diuretics which would certainly be used clinically, as well as the study's “failure to restrict salt” disadvantaged the lisinopril group, especially since “high salt diets minimize the antihypertensive effects” of ACE inhibitors.^20^ Comparatively, 40% of the chlorthalidone (diuretic) group also used the beta-blocker atenolol which “works synergistically with diuretics” (in part, through renin suppression) without any subgroup analysis on the effect of atenolol on the study's outcomes.^20^

The authors of ALLHAT acknowledged the potential faults of their design which “led to a somewhat artificial regimen … of step-up drugs in the ACE inhibitor group,” and “may have contributed to higher BPs in the ACE inhibitor group, especially in the Black subgroup.”^18,^^†^ BP differences can hugely impact risk of stroke as well as other outcomes related to morbidity and mortality.^19^ For stroke (in terms of lisinopril vs. chlorthalidone), the reported RR was 1.40 (95% confidence interval [CI], 1.17–1.68) for Black folks and 1.00 (95% CI, 0.85–1.17) for nonblack folks.^17^

Accounting for the 13–16% difference in RR (that the ALLHAT authors surmised could have been caused by higher SBP in the lisinopril group)^18^ due to an artificial regimen creates a much greater overlap of CIs between groups with a supposedly distinct drug response.

Fourth, the lisinopril versus chlorthalidone stroke RR was not the only example of an overstated difference between Black and nonblack patients in terms of these two drugs. For combined CVD, the RR was 1.19 (95% CI, 1.09–1.30) for Black folks and 1.06 (95% CI, 1.00–1.13) for nonblack folks.^17^ The overlap of the CIs from 1.09 to 1.13 means no statistically significant difference between racial groups can be inferred. Furthermore, HF (the event rates of which were not statistically significantly different for lisinopril vs. chlorthalidone)^‡^ alongside stroke events were two factors largely “responsible for the higher rate of combined CVD in the lisinopril group.”^19^ The validity of information on stroke events was impacted by the artificial study design, and as mentioned earlier, HF was likely variable in its diagnosis due to missing criteria.

Finally, JNC 8 ultimately formulated its racialized recommendation seven through selective uptake of ALLHAT outcomes. It is essential to note that, according to JNC 8, while “a CCB was less effective than a diuretic in preventing heart failure in the Black subgroup of this trial,”^§^ it was still recommended as a first-line therapy for Black patients because “there were no differences in other outcomes (cerebrovascular, CHD, combined cardiovascular, and kidney outcomes, or overall mortality) between a CCB and a diuretic.”^2^

Comparatively, ACE inhibitors were not recommended for Black patients as “a thiazide-type diuretic was shown to be more effective in improving cerebrovascular, heart failure, and combined cardiovascular outcomes compared to an ACEI in the Black patient subgroup.”^2^ Thus, the JNC 8 guideline deems the disparate outcomes between Black and nonblack patients reported by ALLHAT for an ACE inhibitor versus a diuretic as essential enough to discourage the former drug as a first line therapy for Black patients—but not the disparate outcome between Black and nonblack patients for a CCB versus a diuretic.

## The Self-Fulfilling Prophecy of Racial Reasoning

We have shown that the ALLHAT study, and the way it was incorporated into the JNC 8 guidelines, was deeply flawed. ALLHAT produced the idea of a significant difference between Black and nonblack people in drug response and influenced a differential clinical guideline. Given the flaws, we pointed out in its evidence basis, which are not deeply buried or obscure, what explains the persistence of the racialized guideline? In the case of the race-based drug BiDil, Jonathan Kahn has shown the financial interests at play.^22^ We have no reason to think that something similar is at play here, rather we advance a cultural explanation. This is a case of the RWV^23^ becoming a self-fulfilling prophecy in biomedicine.

The RWV was theorized by anthropologist Audrey Smedley as an ideological/cognitive framework that holds that biology is the most important way to understand human differences, and that the groups we think of as racial are primarily biological in nature—while social and economic causes of differences exist, there are crucial underlying biological differences.^23^ The RWV encourages us to think of Black and white as coherent biological categories, to always assume we will find biological differences between them, and to assume that variation within racial categories is relatively trivial while differences between categories are significant. Smedley and others have traced how this worldview emerged and became part of our cultural bedrock^23–26^—even as racial grouping is demonstrated not to be a natural or accurate biological construct.^27–30^

Although science is supposed to be a process by which fallacious ideas are steadily falsified, theoretical frameworks structuring questions, perceptions, and evaluations rarely are overcome with data alone.

The RWV has long preceded biomedical research, but sociologist Steven Epstein has shown how it became concretely institutionalized within biomedicine through the NIH Revitalization Act of 1993.^31^ This Act dictated that the NIH “shall ensure that [trials are] designed and carried out in a manner sufficient to provide for valid analysis of whether the variables being studied in the trial affect women or members of minority groups, as the case may be, differently than other subjects in the trial.”^32^

Essentially, the Act instructed NIH-funded studies (including ALLHAT) to find and publish race-specific medical differences. Crucially though, it was not motivated by racial animus, but rather a push from members of marginalized groups to be represented (nonexploitatively) in biomedical research—a form of racial justice.^31^ But good intentions without a critical perspective on how to think about and analyze racial difference can lead, as they have here, to a treatment paradigm that builds on racist cognition.

In the case of ALLHAT and JNC 8, questions were asked, research was framed, and data were evaluted in a racialized framework, and thus, results were interpreted as confirming the idea that inherent racial differences are driving the inequity in hypertension burden, demanding differential treatment regimen. The RWV becomes a self-fulfilling prophecy^33^ as the assumption of racial difference colors study design, execution, data interpretation, and results presentation in ways that confirm the original assumption. This can be seen across several problems we identified in the previous section. For example, the reliance on secondary outcomes that pointed toward racial difference when the primary outcomes did not do so and the overstated interpretation of overlapping CIs, alongside JNC 8's acceptance of these practices, are puzzling without the overarching search for racial difference conditioned by the RWV.

What underlies these faulty assumptions and animates the RWV in this case? First, is the vague and ambiguous way that race is conceived and operationalized. This has two parts: how groups are identified and defined (considered second) and what race *is*. As Gravlee et al. have argued, in most studies, race is a poorly defined proxy that simultaneously evokes genetic ancestry/heterogeneity; differences in socioeconomic status (SES), opportunities, and health care access; racist treatment; and behavioral and lifestyle differences.^34,35^ This is reflected in ALLHAT and JNC 8, where the racial disparity was marked, but its character or cause never clarified. ALLHAT asserted as its motivation for including Black patients their potential inability to afford drugs aside from diuretics—a SES difference.^7^ But otherwise, blackness is medically undefined and implicitly stands in for a biological difference. Whether that difference is inherent, developmental, or due to later-life environmental factors is never stated.

The other part of the vague invocation of race was the lack of critical interrogation of how racial groups were defined and how individuals were assigned to them. There is no biological coherence to their racial category definitions. ALLHAT used a “self-described Black” subgroup, but blackness is constructed and defined differently among the locations (623 clinics across the United States, the US Virgin Islands, Canada, and Puerto Rico)^18^ from which study participants were recruited. Moreover, the Black populations within each study location are greatly genetically heterogeneous; members are not biologically uniform.^28,36,37^ Of the 19.1% of the study categorized as Hispanic, 65.6% self-identified as “white Hispanic” but were excluded from the “white” category (and placed in the “other” category) in at least one article comparing baseline characteristics of participants in different treatment groups, while the 17.4% of Hispanic participants who identified as “Black Hispanic” were grouped with other Black participants.^38^

No justification was offered for this decision, but it fits with the “one drop rule” for U.S. racial designation which defines whiteness in terms of ancestral “purity” and blackness in terms of any amount of Black ancestry.^30^ Indeed, socially constructed racial grouping was implied to be biological throughout ALLHAT's study design and analysis—and its results seem to imply that disparities in hypertension and drug treatment response are rooted in inherent differential biology between racial groups.

Crucially, however, this appearance may be contingent on who is *not* included in comparisons. Cooper et al. compared rates of hypertension among US white and Black individuals and Caribbean Black individuals, but also included white Germans and Black Nigerians.^39^ Although US Black individuals had greater levels of hypertension than US white individuals, Cooper et al. found that white Germans had the highest and Black Nigerians the lowest. By including a wider range of groups, the hypertension gradient no longer appears to be a function of racial biology, but a more complex pattern of environmental exposures.^39^ Yet, analyzing hypertension solely from the point of view of US-based racial categories yields the appearance of a racial logic to the disparity in disease burden, which is another self-fulfilling prophecy.

There is an implicit framework used to explain this inequity, which is that Black people disproportionately suffer from hypertension because of an evolved predisposition to retain salt.^40^ This so-called the Slavery Hypertension Hypothesis holds that during the slave trade, kidnapped Africans were taken from a homeland of salt scarcity and then experienced the Middle Passage voyage defined by “heat stress and salt and water deprivation” and salt loss from sweat, vomit, and diarrhea.^40^ This “rapid selection process attributed to high mortality during slave transport and slave labor” would, *perhaps*, have led to intense selection for “salt-saving renal-adrenal adaptations” that predisposed African Americans to hypertension.^40^

This connects to the common idea in biomedical research that Black people are predisposed to low concentrations of renin, the enzyme that converts angiotensin to angiotensin I in the RAAS, and therefore decreased PRA.^14^ This is conceptualized within the JNC 8 guideline: ACE inhibitors (which work in the RAAS) are not recommended as an initial therapy for Black hypertensive patients, until they have CKD, and their renin levels are presumably elevated to a level higher than “typical” for Black patients. The influence of the Slavery Hypertension Hypothesis becomes clear upon considering that although a CCB (which does *not* operate within the RAAS) also appeared to be less effective than a diuretic in ALLHAT's Black subgroup, JNC 8 still recommended CCBs as a first-line therapy for Black patients.

The Slavery Hypertension Hypothesis has a specific appeal for health disparities researchers entangled in the RWV. First, it embeds the racialized disparity in hypertension burden and a supposed racialized differential response to ACE inhibitors in an appealing and intuitive evolutionary narrative. The narrative, further, has biopolitical appeal since it explains a racial difference as inherent and the result of natural selection, yet, not as an essential flaw of Black people *per se* but a specific product of past racism. It holds that Black people were “intractably transformed, genetically mutilated,”^40^ which means that contemporary health inequity is not the result of current social forces or discrimination. So, it is a framework that features biological determinism while acknowledging racism but not demanding any antiracist action or social amelioration.

While the ideological logic of the Slavery Hypertension Hypothesis is strong, its evolutionary, genetic, and empirical basis is extraordinarily weak. Critics have shown that theorized patterns do not match contemporary population genetics or the historic landscape of West Africa.^40–43^ PRA levels have been shown to be variable within Black populations.^44,^^**^

A study with a diverse New York City sample investigating PRA variation by age, race, sex, and diabetic status found that, “Despite differences in renin associated with several characteristics, we have found that no demographic factor nor other commonly measured physical or laboratory characteristic, alone or in combination, can be used as a surrogate to predict PRA status. Instead, in all subgroups a wide distribution of PRA values is to be found.”^45^ Yet, Black patients' presentation of low renin alongside salt sensitivity and higher BP has been reported by many sources as inherent.^12,15,44,46^

As we have argued, racial reasoning has a circular logic that is difficult to disrupt. The RWV leads researchers to expect racial differences, and thus studies are designed and interpreted to produce such differences. The hypothesis marshaled to explain racial difference is grounded in the conceptual architecture of the RWV which is itself reinforced by the racial disparity that it produced. When the hypothesis is criticized on theoretical and empirical grounds, one can point to the hypertension disparity as justification that there must be some hidden biological factor to explain the situation. Offering Black and nonblack patients different treatment regimens is a logical outcome, even if the signal of differential drug response is largely a product of the expectation that it exists.

## Discussion

Some practitioners of medicine and public health have tried to sweep away the more radical implications of the social constructionist critique of race by asserting that health inequities are real and need attention and that a practical reliance on progressive ideals can harm patients.^47^ But the racial realism that these folks fall back on as a safer option for protecting patients in fact may do the opposite—demonstrated, for example, by the studies cited earlier that showed differential drug prescription does not assuage disparities in CVD mortality^4^ and that the efficacy of ACE inhibitors on the individual level are not well predicted by the average response of the racial group to which a patient is assigned.^5^

The “inclusion and difference” paradigm ushered in by the 1993 NIH Revitalization Act was preceded by deep concern especially from minoritized populations about scientific exploitation, science performed for the purpose of racial stratification, and complete exclusion from biomedical research. But this paradigm aimed at equity becomes dangerous in its reliance on a spurious assumption of inherent differential biology between racial groups and a mandate of intentional study of these differences to provide responsible treatment, with little focus on social determinants of health. This allows purely genetic explanations for unequal health outcomes to persist.

The slavery hypertension hypothesis, a genetically deterministic explanation of disparity in hypertension burden, for example, does not assign culpability to patients' ongoing lived experiences and blames instead a singular and far-reaching point in history. It absolves practitioners of responsibility in assuaging the structural violence that may create disparate health outcomes between Black and white patients.

Clinical guidelines rooted in this logic of race-based differential biology allow physicians to identify a racial health inequity and locate its purported salvation within the accepted medical milieu, by prescribing Black patients alternative medications. The idea that unequal health care access and the embodiment of the social exposome may be drivers of health disparities is much more disruptive to the RWV than the notion that race-specific drug prescriptions will moderate racialized disparities in CVD. Without the critical tools to conceptualize race effectively, researchers and clinicians can perpetuate pseudoscientific stereotypes with the possibility of medical harm.

The JNC 8 guideline joins many examples of race corrective guidelines and tools being questioned,^48^ rightly so, by practitioners eager for alternative structures through which to treat their patients and grapple with inequity without using circular reasoning to delineate any demonstrated racial variation as due to inherent genetic differences. We advocate for several related approaches; namely, biocritical inquiry,^49^ structural competency,^50^ and race-conscious medicine.^48^ These concepts disrupt the practice of attributing differences in health and treatment outcomes to discrete biological characteristics of different racial groups.

Rather, they encourage an understanding of race as a social product of processes of racialization used to underwrite and justify colonization, dispossession, and enslavement, with effects that persist today.^48^ They contribute to a new scientific paradigm which considers specific genetic determinants of disease and how humans embody their sociopolitical environment, especially lived experiences of racism, and the consequential effects on health outcomes.

Furthermore, structural competency advocates for engagement with interdisciplinary knowledge within medicine and asserts that “increasing recognition of the ways in which social and economic forces produce symptoms or methylate genes then needs to be better coupled with medical models for structural change.”^50^

One powerful example of research informed by a biocritical approach is Dr. Clarence Gravlee's study on determinants of BP in Puerto Rico which finds that African ancestry (via genetic markers) is not associated with high blood pressure (HBP), but that social classification (shaped by skin color) is linked to HBP, through an interaction with SES.^35,51^ For lighter-skinned Puerto Ricans, as SES increases, BP decreases, while the opposite trend is found for ‘Negro’ or dark-skinned Puerto Ricans. It is clear that the environment of ‘Negro’ Puerto Ricans is determined by social classification and SES, which together impact experiences of racism or microaggressions, psychosocial stress, and, ultimately, HBP.^35,51^ Gravlee's methodology is unique for its simultaneous engagement of genetic and social hypotheses.

Many studies, including ALLHAT, fail to use a biocritical inquiry. Without defining or operationalizing race, they use it roughly as a proxy for genetic ancestry, environment, and social treatment, which blocks them from teasing out the specific structural factors that lead to health inequities.

The ALLHAT researchers indicated their buy-in to the RWV as soon as they asserted the importance of a race-specific analysis (so that study results would be applicable to Black patients) in a biomedical research study testing drugs with different mechanisms. Also, JNC 8's rooting in the RWV is demonstrated through its (1) usage of ALLHAT's outcomes as the evidence basis for racialized recommendations despite its clearly problematic study design and analysis; (2) assumption, without genetic evidence or further investigation of other variables, that racial differences in the study were the product of differential biology that indicates distinct drug treatment based on race; and (3) selective uptake of outcomes to create guidelines aligned with historical notions of a Black patient-specific physiology characterized by low renin and salt sensitivity.

The self-fulfilling prophecy of racial reasoning is apparent here as the JNC 8 guideline further entrenches notions of immutable differential biology between racial groups which permeate biomedical research and clinical treatment. As Dr. Camara Jones puts it, “The ideology of race as a biologic determinant is bolstered when scientists fail to probe the basis of race-associated differences as though this basis were already completely understood.”^52^ Gravlee demonstrates that alternative approaches grappling with genetic and environmental factors are necessary for researchers and clinicians to measure, investigate, and understand the specific aspects of a person or group's environment that impact their health outcomes so that these can be mitigated.

In accordance with other scholars,^48^ we assert that the medical and public health fields need to look carefully at their practices and reevaluate previous findings through biocritical inquiry and the consideration of the impact of mediators, including structural factors, on commonly propagated racialized findings. We advocate for the incorporation of the highest standards of evidence and self-critical reflexivity to ensure that researchers and clinicians are not inscribing the RWV falsely into medicine.

Notably, we are not suggesting a colorblind approach that ignores the impacts of racialization and racism on health outcomes. Rather, we believe that racial health inequities need to be addressed through a lens of justice. We champion a rejection of the status quo of racial realism that may only exacerbate inequity, and an embrace of structural competency and race-conscious medicine. We push for the conceptualization of the process of racialization as a tool of colonization and social stratification, the effects of which can become embodied through structural violence that clinicians and researchers have a responsibility to address.

## Abbreviations Used

ACE: angiotensin-converting enzyme
ALLHAT: Antihypertensive and Lipid-Lowering Treatment to Prevent Heart Attack Trial
ARB: angiotensin receptor blocker
BP: blood pressure
CCB: calcium channel blocker
CHD: coronary heart disease
CI: confidence interval
CKD: chronic kidney disease
CVD: cardiovascular disease
HBP: high blood pressure
HF: heart failure
JNC: Eighth Joint National Committee
PRA: plasma renin activity
RAAS: renin-angiotensin-aldosterone system
RR: relative risk
RWV: Racial Worldview
SBP: systolic blood pressure
SES: socioeconomic status

## Notes

^*^
It is out of the scope of this article to develop this point further; we want to acknowledge that this may be an oversimplification.

^†^
For SBP, after 1 year, in the Black subgroup, lisinopril decreased SBP, on average, 2.5 mmHg, compared to 7.7 mmHg with chlorthalidone. In the nonblack subgroup, lisinopril decreased SBP 8.1 mmHg after 1 year compared to 9.8 mmHg for chlorthalidone.

^‡^
RR was 1.30 (95% CI, 1.10–1.54) for Black patients, 1.13 (95% CI, 1.00–1.28) for nonblack patients.

^§^
For amlodipine versus chlorthalidone, there was a 1.46 RR (95% CI, 1.24–1.73) of HF for Black participants and 1.32 RR (95% CI, 1.17–1.49) for nonblack participants. Importantly, although the relative risks are distinct, these confidence intervals overlap from 1.24 to 1.49.

^**^
Cited PRA levels often refer to systemic rather than tissue renin activity, the latter of which is more impactful in hypertension-related organ damage.^14^
